# Research Progress in Strategies for Enhancing the Conductivity and Conductive Mechanism of LiFePO_4_ Cathode Materials

**DOI:** 10.3390/molecules29225250

**Published:** 2024-11-06

**Authors:** Li Wang, Hongli Chen, Yuxi Zhang, Jinyu Liu, Lin Peng

**Affiliations:** School of Chemistry and Chemical Engineering, Hebei Minzu Normal University, Chengde 067000, China

**Keywords:** lithium iron phosphate, conductivity, carbon coating, doping

## Abstract

LiFePO_4_ is a cathode material for lithium (Li)-ion batteries known for its excellent performance. However, compared with layered oxides and other ternary Li-ion battery materials, LiFePO_4_ cathode material exhibits low electronic conductivity due to its structural limitations. This limitation significantly impacts the charge/discharge rates and practical applications of LiFePO_4_. This paper reviews recent advancements in strategies aimed at enhancing the electronic conductivity of LiFePO_4_. Efficient strategies with a sound theoretical basis, such as in-situ carbon coating, the establishment of multi-dimensional conductive networks, and ion doping, are discussed. Theoretical frameworks underlying the conductivity enhancement post-modification are summarized and analyzed. Finally, future development trends and research directions in carbon coating and doping are anticipated.

## 1. Introduction

Currently, the research and development of high-energy-density cathode materials is a crucial focus within battery material science, with a parallel emphasis on battery safety. Since its initial report in 1997, olivine-type LiFePO_4_ has emerged as a leading contender in power battery and energy storage applications due to its exceptional thermal stability and safety [1,2,3,4]. However, inherent structural limitations impede the free diffusion of electrons and Li ions within the LiFePO_4_ olivine framework. Notably, Li-ion movement along the c-axis is obstructed, restricting ions to a non-linear zigzag pathway along the b-axis. Because of the structural limitations, LiFePO_4_ exhibits low electronic conductivity and Li-ion diffusion coefficients, hampering its potential for achieving high energy density and rapid charge–discharge performance [5,6,7]. A key strategy to enhance the electrochemical performance of LiFePO_4_ is carbon coating, which involves enveloping LiFePO_4_ particles with a carbon layer and interconnecting them with a conductive carbon network. This approach significantly enhances the external conductive environment of the particles, thereby enhancing the electrochemical performance of the material. Additionally, the presence of carbon materials effectively inhibits the growth of particles, creating excellent conditions for particle nanoization. Different from surface coating, ion doping involves introducing metal or non-metal ions into various positions within the LiFePO_4_ structure to reduce band gap width, generate lattice defects, change semiconductor properties, broaden ion transport pathways, add ionic conductive materials, construct defects in carbon layers, etc. [8,9,10,11,12,13,14]. Thus, this review highlights recent advancements in enhancing the conductivity of LiFePO_4_ materials, encompassing strategies such as in situ carbon coating, the establishment of multi-dimensional conductive networks, and ion doping.

## 2. The Effectiveness of Modification and Ion Doping

In the stable internal spatial structure of lithium iron phosphate, electrons and lithium ions are difficult to diffuse and shuttle freely, the movement of ions in the c-axis direction is hindered, and they can only perform a non-linear sawtooth-like shuttle motion in the b-axis direction [1]. Furthermore, relevant theoretical calculations have shown that lithium iron phosphate is a semiconductor with low electronic conductivity [12]. The above shortcomings completely limit the large-scale application of lithium iron phosphate, and are also the theoretical basis for surface modification and internal doping of lithium iron phosphate. In power batteries and energy storage devices, it is also necessary to consider the kinetic characteristics and thermodynamic stability of the particle transport process [15,16,17]. Through thermodynamic and kinetic simulation analysis, materials can be purposefully designed. The strategy for modifying and doping materials requires the selection of efficient and effective implementation methods and characterization methods. Compared to traditional simple physical mixed calcination carbon coating, in situ nanoparticle growth carbon coating, the construction of a multi-dimensional conductive system, and ion doping will be emphasized in this review.

### 2.1. Strategy for Surface Modification

#### 2.1.1. In Situ Carbon Coating

Carbon coating involves applying a layer of carbon material with excellent electrical conductivity onto the surface of LiFePO_4_ particles using various methods. This enhances the electronic conductivity between particles and stabilizes the coated cathode material during electrolyte and electrochemical reactions [18,19]. The carbon sources utilized include organic and inorganic carbon materials, carbon fibers, and carbon nanomaterials [20,21,22,23,24]. In addition to improving the electron conductivity, a uniform carbon layer on nanoparticles prevents uneven conduction due to material agglomeration.

The in-situ carbon coating method yields superior coating results by significantly enhancing particle-to-collector fluid contact, thus improving electron conductivity. The introduction of the carbon source before particle formation prevents particle growth during high-temperature sintering, controlling particle size and enhancing material electrochemical activity [25]. In situ carbon coating can be understood as two aspects: (1) the in-situ growth of LiFePO_4_ particles on the surface of carbon materials (such as graphene and carbon nanotubes) [26,27]; (2) the in-situ growth of carbon-containing materials on the surface of LiFePO_4_ [21]. Regardless of the selection of raw materials, the purpose of in situ coating is to achieve chemical bonding between carbon and LiFePO_4_, achieving good conductivity.

Graphene is a typical graphite carbon structure material which has a regular layered carbon structure and can construct an excellent 2D conductive carbon network and elastic structure [28]. The distinctive two-dimensional configuration, irregular surface topography, impurities from different atoms, enhanced contact between the electrode and the electrolyte, augmented spacing between layers, and heightened electrical conductivity all contribute to swift surface lithium-ion absorption and extremely rapid lithium-ion diffusion along with electron transfer [29,30].

**Figure 1:**
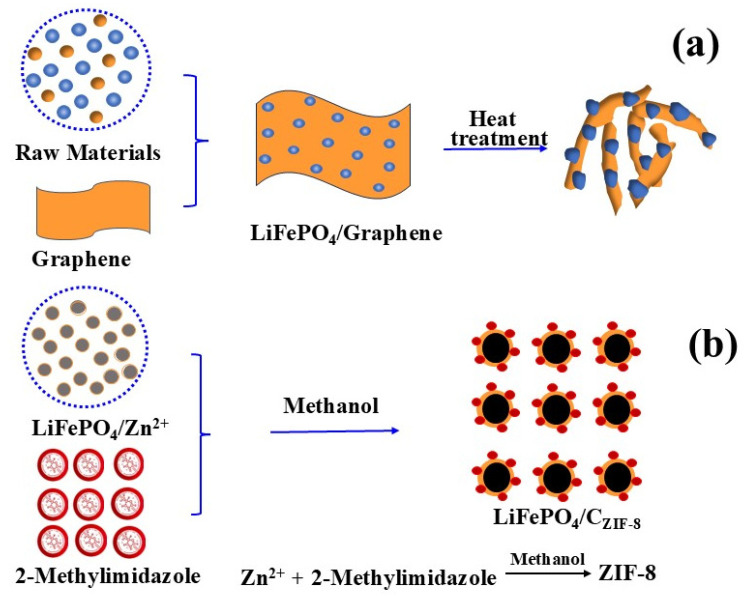
(**a**) Schematic image of LiFePO_4_ growth on unfolded graphene [26]. Adapted from [26]. (**b**) Schematic image of C_ZIF-8_ growth on the surface of LiFePO_4_ [31]. Adapted from [31].

Yang [26] studied an in-situ growth method, growing LiFePO_4_ nanoparticles on monolayer graphene with excellent dispersion (Figure 1a). Monolayer graphene provides a high-quality three-dimensional (3D) conductive network, enabling each LiFePO_4_ particle to attach to the conductive layer (Figure 1a). This method substantially improves material electrical conductivity, leading to enhanced electrochemical properties. The initial discharge capacity reached 166.2 mAh g^−1^ (98% of theoretical value).

**Figure 2:**
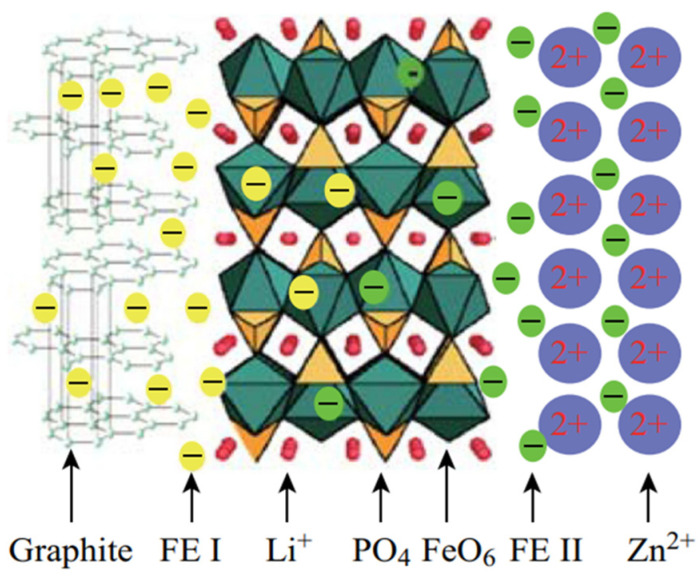
Mechanism of conductivity improvement. FE I represents free electron in graphite; FE II is free electron in metal zinc [31]. Reprinted from [31].

Xu [31] studied the in-situ coating of zeolite-imidazole ZIF-8 on commercial LiFePO_4_ material with a thickness of ~10 nm. The study analyzed coating structure and metal zinc (Zn) distribution on the LiFePO_4_ (LFP) surface (Figure 1b). Nucleation and crystal growth of ZIF-8 nanoparticles on LiFePO_4_ surfaces were followed by new graphite-like carbon appearance and generation post-calcination. The results indicated that the LFP/C_ZIF-8_ material exhibited a heterogeneous electrical conductivity mechanism, and the graphitic carbon in the material exhibited exceptional electrical conductivity because of the ordered sp^2^ carbon and free electrons (yellow sphere, FE I in Figure 2). An optimal carbon coating material should maximize free electrons, facilitating inter-regional electron flow to enhance electrochemical material properties.

The carbon coating process represents material surface modification. The carbon layer serves as an interface between the cathode material and electrolyte, facilitating electron and Li-ion transfer crucial to material performance. The binding force between C and LiFePO_4_ and the mechanism of enhanced conductivity have become a focus of attention after carbon coating.

**Figure 3:**
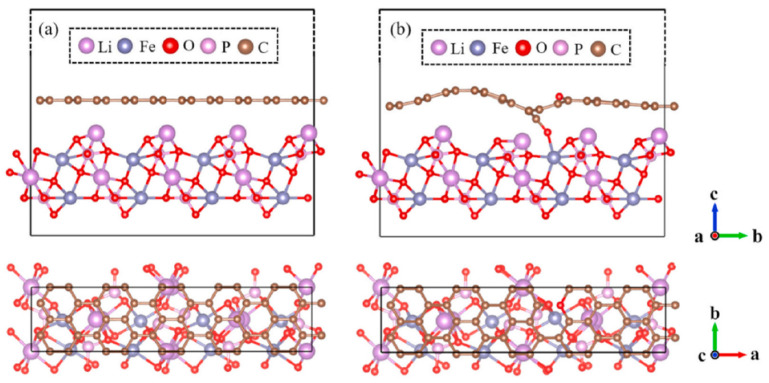
The relaxed atomic structures of (**a**) LFP/G and (**b**) LFP/GO [32]. Reprinted from [32].

Recent studies have elucidated how defective graphene oxide (GO) coating enhanced LiFePO_4_ conductivity through theoretical calculations. Chen [32] investigated the electronic structure of GO parallel to the LiFePO_4_ surface using first-principles density functional theory calculations within the DFT+U framework. The results indicated that the emergence of bands in gap states originated from graphene coating. Furthermore, GO was attached to LiFePO_4_ (010) through C-O and Fe-O bonds, instead of the attraction of van der Waals forces. The chemical bonds (Fe-O-C) are shown in Figure 3. Thus, the LFP/GO interface facilitated the electronic conductivity of the interface.

**Figure 4:**
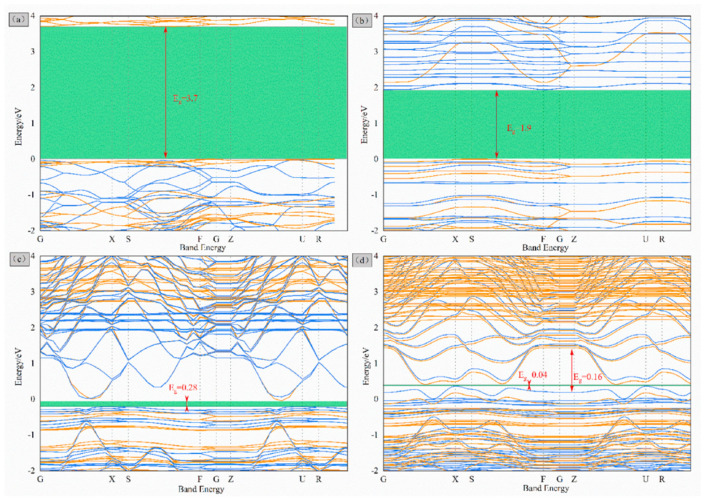
Band structure at the Fermi level for (**a**) LFP bulk, (**b**) LFP (010), (**c**) G on LFP (010), and (**d**) GO on LFP (010) [32]. Reprinted from [32].

The electronic energy band calculations (Figure 4) indicate increased density in valence and conduction bands owing to GO interaction with LiFePO_4_ (010), indicating Fe-O-C bond existence. This review elucidates the principle and advantages of in situ carbon coating, enhancing the surface conductivity of LiFePO_4_ materials, and offering theoretical support for carbon coating modification of other similar materials.

Graphite carbon has excellent electrical conductivity, which is why researchers choose this type of material for carbon coating. In addition to graphene, other carbon materials can also be processed to achieve graphite carbon, and their conductivity can be optimized through improvements in the manufacturing process. Apart from graphene, other carbon materials can also be processed to obtain graphite carbon, and their electrical conductivity can be maximized through advancements in the manufacturing process. Raman analysis can be used to detect the degree of graphitization of the carbon layer coated on the surface of materials. The use of this technique makes the design of materials more superior [33,34].

In summary, the in-situ carbon coating strategy is an excellent method for enhancing the surface and conductivity of materials. However, before implementing carbon coating, a detailed analysis and explanation of the conductive mechanism should be conducted to enhance coating material design and structural optimization. In situ carbon coating holds promise for modifying surface conductivity in other insulators or semiconductor materials, achieving dual electronic conduction and material application effects.

#### 2.1.2. Surface Carbon Layer Doping and LiFePO_4_ Modification

In research on some non-in situ carbon coating processes, researchers have devoted considerable effort to doping the carbon layer with non-metal atoms. The main non-metal elements used and their primary functions in carbon layer doping are listed in Table 1 below.

It was reported that additional electrons contributed by the N atom can provide electron carriers for the conduction band, which can contribute to the electrical conductivity of the material by introducing N into the carbon structures [35,36]. The F atom has a higher electronegativity than other anions, and F doping will accelerate the decrease in the interfacial resistance of the battery [37]. Coating lithium iron phosphate with sulfur-doped graphene nanosheets can create an electronic conductive network and can also promote the transportation of electrons and Li-ions [38,39,40]. Phosphorus-doped carbon layers can decrease transfer resistance and are good at the graphitization of the carbon [41]. The multi-element doping of carbon layers can achieve higher electronic conductivity and lower migration activation energy [42]. The main function of carbon coating and carbon layer doping is to enhance the electronic conductivity of the material. Investigations have demonstrated that the application of electrochemically active electron-conducting polymer coatings on LiFePO_4_ particles could potentially replicate the roles of carbon coatings, while also being applicable under less stringent conditions and offering the extra benefit of improved ionic conductivity within the active material [44,45].

Data from Table 1 demonstrate that the electrochemical performance and electronic conductivity of lithium iron phosphate (LFP) materials are significantly enhanced after doping the carbon layer with certain non-metal elements. By doping the carbon layer with heteroatoms such as nitrogen (N), sulfur (S), and boron (B), the conductivity of the carbon layer can be increased. For example, nitrogen doping can introduce additional electrons, thereby enhancing the electronic conductivity of the carbon layer. On the other hand, non-metal elements can assist in forming a conductive network within the carbon layer: doping with heteroatoms can promote the formation of a more comprehensive conductive network, thereby increasing the electron transfer rate of the electrode material. Furthermore, doping the carbon layer can improve the ion diffusion pathways: doping with heteroatoms can alter the surface structure of the material, providing lithium ions with shorter diffusion paths and thus enhancing the migration rate of ions. For instance, co-doping the carbon layer with nitrogen and boron can greatly enhance the electrochemical performance: at a rate of 20 C, the co-doped sample can increase the discharge capacity of LFP/C from 101.1 mAh g^−1^ to 121.6 mAh g^−1^ [42].

#### 2.1.3. Ion Conductive Materials

Charging and discharging reactions of a battery are the result of the combined migration of ions and electrons. Especially during high-current charging and discharging, it is necessary to consider both the transport of electrons and the migration of ions. If the combined effects of both can be taken into account comprehensively, it will greatly enhance the electrochemical performance of the material. The main ways to improve the electrical conductivity include coating and modification with conductive carbon materials on the surface and doping with ions inside the material. To increase the migration rate of lithium ions, it is common to reduce the particle size of the material and construct special structures [46,47].

The ion conductive materials can enhance the electrochemical performances of LiFePO_4_ because of their high ionic conductivity and lithium ionic storage ability. Typically, graphene is often regarded as an excellent electronic conductor which can significantly enhance the electronic conductivity on the surface of LiFePO_4_. Most layered structures of graphene without defects would hinder the transition of Li^+^ [48]. The enhancement of ionic conductivity requires more attention. Incorporating GO (graphene oxide) contributes to the preservation of material stability and the augmentation of lithium ion diffusion coefficients since the lithium ions have lower insertion and extraction potentials along the [010] facet in LiFePO_4_ [32,49]. After being coated with graphene or GO, the average Fe-O bonds on the LiFePO_4_ (010) surface underwent significant changes, which led to the expansion of the Li^+^ channel, facilitating the insertion and extraction of migrating Li^+^. In addition to the lithium ions provided by LiFePO_4_ itself, incorporating lithium-ion conductive materials into the material can provide additional support for the intercalation and deintercalation of lithium ions, which will greatly enhance the material’s rate performance and cycling performance. Some ion conducting materials have a special three-dimensional structure that can facilitate rapid diffusion of Li^+^ [50]. Chien [51] designed a LiFePO_4_/Li_3_V_2_(PO_4_)_3_/C composite cathode material to help enhance the diffusion properties of lithium ions. Essentially, the enhancement of ionic conductivity of coated materials is contingent upon their unique post-modification structures. Materials that facilitate lithium-ion conduction possess both high ionic conductivity and lithium-ion storage capability, thereby significantly boosting the ionic conductivity of LiFePO_4_.

### 2.2. Strategy for Building a Multi-Dimensional Conductive Network

Nanoparticles of LiFePO_4_ are expected to be used as the cathode material of high-performance lithium-ion batteries [52]. The design and preparation of nanocomposites can effectively improve the low thermal stability and multiple surface side reactions of nanoparticles [53]. The design and the construction of conductive network structures have been the main focus of research in recent years [27,54,55,56,57,58,59,60]. Constructing a multi-dimensional conductive network is one of the effective means to enhance the electronic conductivity of LiFePO_4_. The construction of conductive networks generally uses one-dimensional materials [27] and two-dimensional materials [61] to provide a conductive skeleton in combination with two-dimensional methods. LiFePO_4_ is then used to gradually fill the surface and interior of the skeleton, achieving multi-dimensional conductive effects. A typical multi-dimensional conductive network is shown on Table 1. The main network structure could include a porous structure [54], a hierarchically porous structure [59], a highly meso-porous structure [60], a 3D conducting network [56], a distinctive loose and scaffolded structure [27], etc.

Typical conductive network images are shown in Table 2.

Data from Table 2 clearly indicate that the construction of a 3D conductive network significantly enhances the electrical conductivity and high-rate discharge capacity of lithium iron phosphate (LFP) materials. The excellent electrochemical performance is attributed to the following factors: (i) increased contact between particles, enhancing the efficiency of electron transport within the material; (ii) providing a larger surface area for electrochemical reactions; (iii) allowing more lithium ions to participate in the charging and discharging process. The following text provides a more detailed narrative.

**Figure 5:**
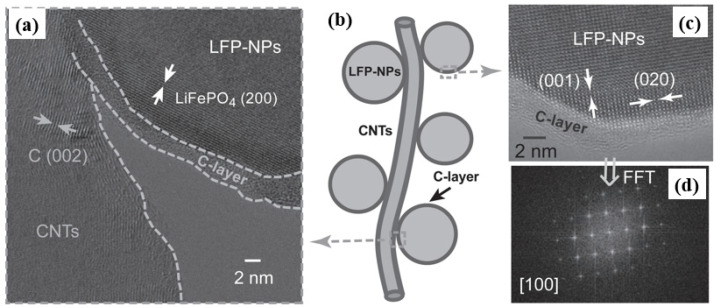
(**a**,**c**) HRTEM images and (**b**) a schematic illustration of the prepared LFP@C/CNT nanocomposite. (**d**) The corresponding FFT of the HRTEM in (**c**) [63]. Reprinted from [63].

Wu [63] designed a new LiFePO_4_ nanoparticle, which exhibited two types of carbon complexes, including amorphous carbon coating and a graphitized conductive structure (Figure 5). Furthermore, compared with the original LFP@C and LFP/CNT coatings, the initial carbon layer evenly coated all nanoscale LiFePO_4_ particles due to the synergistic effect of amorphous carbon. This stabilized the interface of LiFePO_4_ nanoparticles, thereby enhancing electrical conductivity and Li diffusion.

**Figure 6:**
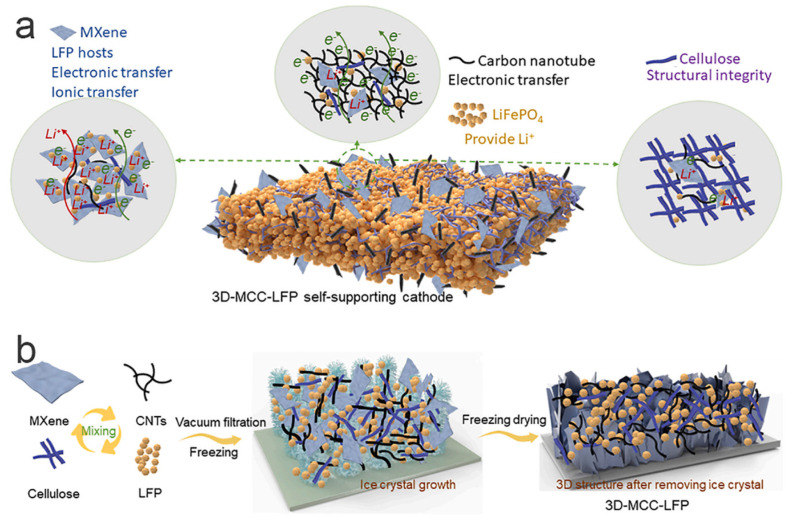
Schematic illustration of the structure (**a**) and fabrication process (**b**) of 3D-MCC-LFP cathode [61]. Reprinted from [61].

Dong and colleagues [61] designed a 3D-MCC-LFP material with a high load rate, exceptional mechanical properties, and excellent electrical conductivity using an assembly method (Figure 6). In this structure, 2D MXene served as a key component, providing sites for LiFePO_4_ particle loading, connecting materials, and facilitating simultaneous electron and ion transport. One-dimensional carbon nanotubes served as conductive agents, enabling full interconnectivity of the scaffold and thereby enhancing electronic conductivity. One-dimensional cellulose served as a reinforcing filler, preserving the mechanical properties and structural integrity of 3D-MCC-LFP while accommodating a large amount of LiFePO_4_ material.

The author employed SnO_2_-NF as the negative electrode material to test the performance of the assembled battery, and due to its excellent conductivity and high loading capacity of LiFePO_4_, the electrochemical properties of 3D-MCC-LFP materials surpassed those prepared using traditional methods.

**Figure 7:**
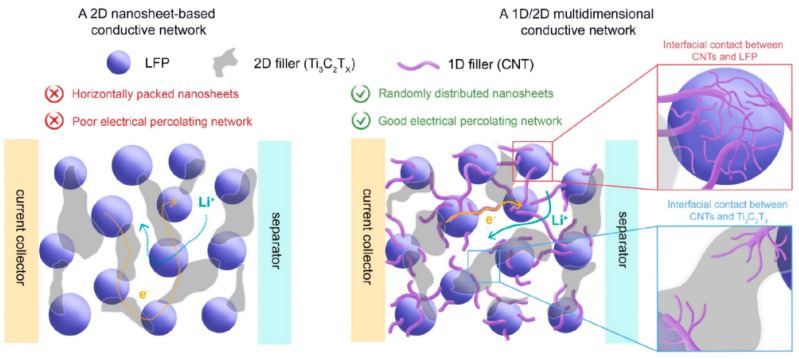
Schematic illustration depicting electrode characteristics in a 2D nanosheet-based conductive network (**left**) and a 1D/2D multi-dimensional conductive network (**right**) with inset schemes showing magnified views of the interfacial contacts between CNTs and LFP particles (**top**) and between CNTs and Ti3C2Tx (**bottom**) in the multi-dimensional conductive network [62]. Reprinted from [62].

Similarly, Checko [62] designed a new multi-dimensional network to enhance local electrical transport across the LiFePO_4_ surface (Figure 7). This structure is consistent with single-walled carbon nanotubes (1D) and MXene nanosheet (2D) bound together. The CNTs facilitated local electron transport across the LiFePO_4_ surface, while Ti_3_C_2_T_x_ nanosheets provided conductive pathways through the bulk of the electrode. The electrochemical characterization supported by numerical simulation verified the charge transfer characteristics of this multi-dimensional conductive network.

Contrastingly, Luo [64] exploited N-doped graphene (NG)-modified LiFePO_4_ material with a 3D conductive network structure for Li-ion batteries. In this structure, NG effectively coated and connected LiFePO_4_ particles, and N doping reduced electrode polarization, enhancing electrochemical reaction reversibility. The special structure constructed with NG provided faster and more efficient 3D transport channels for Li^+^ and electrons.

The construction of a multi-dimensional conductive network is an efficient approach for rapid transmission on and between particle surfaces. The dual connected channel composed of a solid phase network channel and an internal cavity channel with a conductive network structure achieves rapid electron and ion transport, and also ensures uniform contact between the electrolyte and the positive electrode, thereby forming a good interface. The porous structure can achieve effective infiltration of electrolytes, which is beneficial for improving the lithium diffusion rate and reaction kinetics. However, critical factors to consider include the mechanical stability of the structure, electronic transmission efficiency, and compatibility between the conductive network and the material interface.

In addition to intrinsic material properties, achieving excellent carbon coating is essential to fully utilize electrochemical performance. A uniform and effective carbon skeleton conductive network should form between particles, emphasizing molecular-level mixing of carbon skeleton and LiFePO_4_. Conventional physical coating methods with simple processes and low costs may struggle to achieve accurate carbon coating requirements. Therefore, they have become a prospective technology to study new carbon coating methods with controlled nano-growth mechanisms.

### 2.3. Strategy for Ion Doping

**Figure 8:**
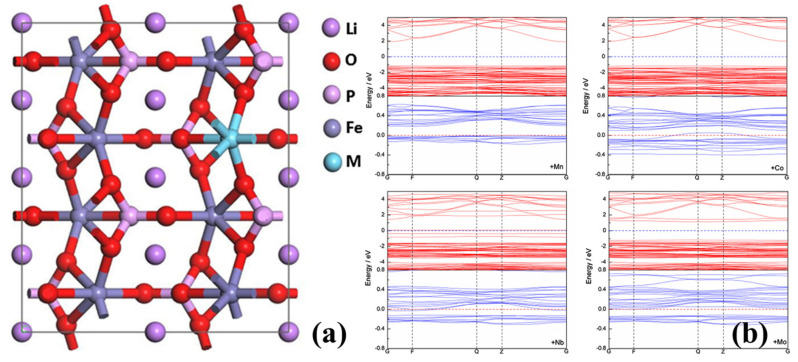
(**a**) Bulk model of M-doped LiFePO_4_ (M = Mn, Co, Nb, Mo). (**b**) Band structures of LiFePO_4_. The red and blue lines represent majority spin states and minority spin states, respectively (for interpretation of the references to color in this figure legend, the reader is referred to the web version of this article) [78]. Reprinted from [78].

The purpose of in situ carbon coating and the construction of a multi-dimensional conductive network on particle surfaces is to enhance electrical conductivity both within and between particles, representing a physical modification of battery materials. However, ion doping constitutes a chemical modification aimed at enhancing intrinsic electrical conductivity [65,66]. Specific doping locations include iron (Fe), phosphorus (P), and Li sites, among which Fe-site doping is the most prevalent [66,67,68,69,70,71]. Additionally, various doping elements have been studied, including manganese (Mn), nickel (Ni), niobium (Nb), magnesium (Mg), cobalt (Co), vanadium (V), and others [72,73,74,75,76,77]. However, Fe-site doping serves a dual purpose. First, it narrows the band gap between the conduction and valence bands of semiconductor material (LiFePO_4_), thereby increasing material conductivity. Second, ion doping induces Li or Fe vacancies, forming charge compensation defects, and the conductivity of electrons is enhanced. Zhang [78] studied the electronic properties of LiFePO_4_ doped with Mn, Co, Nb, Mo, and other elements using first-principles calculations. The results (Figure 8) indicated reduced band gaps with the doping of these elements, facilitating electron transitions. Notably, Co and Nb doping exhibited obvious enhancement effects. Moreover, studies have shown that doped materials inhibit microcracks, prevent electrode polarization, and enhance overall material performance. Furthermore, doping with Mn, Nb, Mo, and Co also enhances the mechanical stability of LiFePO_4_, altering parameters such as material structure, M-O (metal–oxygen) bond energy, and band gap width, thereby enhancing both electrical and mechanical properties.

Dou [12] examined the electronic structure of LiFexMn_1−x_PO_4_ doped with different Mn contents using first-principles density functional methods. The study found that LiFe_0.75_Mn_0.25_PO_4_ exhibited the smallest bandgap width with a Mn doping amount of x = 0.25. This was attributable to the Fe3d electron contributions dominating the density of states (DOS) near the Fermi plane of LiFePO_4_, while Mn3d electrons become predominant upon Mn incorporation, increasing the DOS near the Fermi level in LiFe_0.75_Mn_0.25_O_2_ and enhancing material conductivity. Conversely, introducing Mn lengthened Fe-O bonds and weakened Fe-O bond energy, widening Li-ion migration channels and facilitating ion diffusion.

Ban et al. [79] studied the co-doping of “donor-acceptor” charge compensation, combining theoretical calculations with experiments to significantly enhance material rate performance. It was observed that P and O sites co-doped with silicon (Si) and fluorine (F) altered the conduction band edge of LiFePO_4_, enhancing material conductivity by at least two to three orders of magnitude compared with pre-doping levels. This approach facilitated the positive magnification performance of LiFePO_4_.

In addition to theoretical studies, replacing and occupying the spatial structure of an element in LiFePO_4_ using doping elements to create lattice defects broadens ion transport channels, enhancing material intrinsic conductivity. Marnix [80] studied the doping of hypervalent ions in LiFePO_4_, observing that the ions that occupied Li positions maintained a positive bivalent state in Fe, thus contributing to improved material conductivity.

Recently, the doping of rare earth elements into positive electrode materials has also been widely studied [81,82]. The ionic radius of rare earth elements is larger than that of transition metal elements, resulting in an increased material cell volume and a reduced band gap after doping with rare earth elements. This increase in mobility and carrier concentration significantly enhances the electrical conductivity of the final material [83,84]. Studies have shown that doping Li-ion phosphate with rare earth element ions such as erbium (Er^3+^), yttrium (Y^3+^), and Nd^3+^ leads to Fe replacement by rare earth ions, resulting in increase in material conductivity of four orders of magnitude. This occurred because the electron-deficient rare earth element ions created holes that readily exited full electrons to the hole level, transforming the material from a N-type to a P-type semiconductor [85,86]. Qiu Peng [87] studied the effects of lanthanum (La), Nd, and Y doping on the structure and electrochemical properties of LiFePO_4_. The results indicated increased cell parameters and volume, smaller particle size, and uniform, tightly bonded pores in the doped material. In comparison with pure Li–Fe phosphate, the electrical conductivity of the doped material increased by four orders of magnitude, owing to enhanced Li-ion diffusion facilitated by the small particle size and internal lattice defects created by the doped elements. Studies have shown that rare earth element-doped materials exhibit smaller particle sizes [88,89,90,91,92,93].

Notably, enhancing the diffusion performance of Li-ions is also an important purpose of doping, and its double effect was achieved through the co-doping method [94,95]. Wang [96] successfully synthesized Y-F co-doped LiFePO_4_/C material using a high-temperature solid-phase method. The introduction of F enhanced the electron-cloud rearrangement of PO_4_^3−^, significantly enhancing conductivity. Simultaneously, Y was introduced into Li^+^ vacancy, reducing spatial resistance to Li-ion diffusion and comprehensively enhancing material ionic conductivity. Additionally, X-ray diffraction analysis results revealed weakened Li-O bonds and widened Li-ion diffusion tunnels due to Y-F doping, leading to Li-ion diffusion rates. As a result, the material exhibited excellent electrochemical properties, that is, its specific discharge capacity reached a 179.3 mAh·g^−1^ capacity at a 0.1 C current density and a 135.5 mAh·g^−1^ capacity at 10 C.

The doping method theoretically optimized the structure of the crystal, and the conductivity of the material was fundamentally improved. However, the mechanism by which doping changes the electrochemical properties of materials remains unclear. The electronic conduction within the crystal of the material was very complicated, and whether the doped element fulfilled its designed role remains uncertain, with assessments largely based on macro-level performance. The particle size reduction following rare earth element doping lacks detailed analysis, indicating a partial absence of a relevant theoretical basis. Therefore, careful selection of doping elements and the acquisition of necessary theoretical support are essential prerequisites for material doping research.

## 3. New Carbon Coating Technologies and LiFePO_4_ Batteries

Some new carbon coating technologies are rapidly developing which not only apply to lithium iron phosphate materials but also provide new ideas for surface coating modification of other materials with poor conductivity.

Flash Joule heating (FJH): Using an ex situ carbon coating method, the precursor could rapidly decompose through flash Joule heating (FJH) technology. By depositing carbon heteroatom materials within a limited space in just 10 s, a uniform amorphous carbon layer can be obtained on LFP; at the same time, different heteroatoms can be introduced into the surface carbon layer. Solvent-free, versatile cathode surface modification is the highlight of this technology [97]. Supercritical CO_2_-enhanced surface modification: Employing supercritical CO_2_ (SCCO_2_) for a proficient ex situ carbon coating method on lithium iron phosphate (LFP) results in a superior carbon coating layer with a higher graphite carbon content and reduced oxygen-derived functional groups, significantly improving electron transfer efficiency [98]. Spray coating technology: Using spray coating technology to produce LiFePO_4_-coated carbon fiber (CF) as a structural battery cathode component can yield substantial electrochemical performance for the battery. The use of spray coating technology stands out as an innovative method for manufacturing electrodes in structural batteries, highlighting its capacity to enhance the efficiency of multifunctional energy storage systems [22]. Ultrafast nonequilibrium high-temperature shock technology: This technology introduces Li-Fe anti-site defects and controllable tensile strain into the LiFePO_4_ lattice. This design allows for research on the impact of strain fields on performance to extend from theoretical calculations to experimental perspectives [99]. Recycling and reuse technology for spent LiFePO_4_: This is a direct regeneration of LiFePO_4_ based on a doping strategy, a highly efficient additive for direct reactivation of waste LiFePO_4_, prilling, and a cocoating collaborative strategy [100,101,102].

## 4. Conclusions

The carbon layer structure significantly affects the conductivity of LiFePO_4_. To achieve optimal electrochemical performance, it is necessary to incorporate highly graphitized carbon (sp^2^ hybrid state) to enhance its conductivity. However, traditional organic or inorganic carbon struggles to achieve a high degree of graphitization at sintering temperatures of 500–800 °C. Therefore, while in situ growth of LiFePO_4_ on a single layer of graphene proves promising, the development and adoption of this technology must overcome challenges related to cost and synthesis methods. Furthermore, the quest for new carbon materials with enhanced graphitization remains imperative. Additionally, modifying crystal structures through cationic or anionic doping offers optimization potential. However, the mechanisms and effects of doping reactions on material properties require more comprehensive investigation. The analysis of substitution sites and doping quantities necessitates refined proof methods. Finally, future efforts concerning LiFePO_4_ and other types of cathode (anode) materials with poor conductivity must be focused on achieving high energy and vibration densities alongside stable cycling, excellent rate performance, and low-temperature capabilities. This trajectory underscores the imperative for ongoing advancements in material science and electrochemistry.

## Figures and Tables

**Table 1:** Elements used to dope the carbon layer and their primary functions.

Materials	Doped Atoms	Functions	Discharge Capacity (High Rate)	Conductivity/ ×10^−2^ S cm^−1^	Ref.
Egg white (1 mL)	N	offer superior electronic transportation between LiFePO_4_ active particles	120 mAh g^−1^ (LFP/C+N at 5 C) 113 mAh g^−1^ (LFP/C at 5 C)		[35,36]
Polyvinylidene fluoride (5% wt)	F	play a vital role in the improvement of electron transfer kinetics	121.5 mAh g^−1^ (LFP@FC-II at 10 C) approaching 120.0 mAh g^−1^ (LFP at 0.1 C)		[37]
Sulfur-doped graphene sheet	S	promote the transportation of electrons and Li-ions; prevent volume change during the Li^+^ intercalation/deintercalation procedure	130.5 mAh g^−1^ (LiFePO_4_@C/S-doped graphene at 10 C) 116.5 mAh g^−1^ LiFePO_4_@C		[38,39]
Oxalic acid and benzyl disulfide	S	promote the electronic conductivity and defect level of the carbon	137 mAh g^−1^ (LiFePO_4_/SC at 5 C) 128.5 mAh g^−1^ (LiFePO_4_/C at 5 C)		[40]
Triphenylphosphine (0.2 g mL^−1^ was mixed with 4 g LiFePO_4_/C)	P	benefit the graphitization of the carbon; decrease transfer resistance	124.0 mAh g^−1^ (LFP/C-P3 at 20 C) 105.4 mAh g^−1^ (LFP/C at 20 C)		[41]
Melamine, boric acid	N+B	electron-type and the hole-type carriers donated by nitrogen and boron atoms generate the synergistic effect to greatly elevate the high-rate capacity	121.6 mAh g^−1^ (LFP/C-N+B at 20 C) 101.1 mAh g^−1^ (LFP/C at 20 C)	13.6 (LFP/C-N+B) 2.56 (LFP/C)	[42]
Methionine	S+N	good ionic and electronic conductivities	103 mAh g^−1^ (NSC@LFP at 2 C) 63 mAh g^−1^ (pristine LFP at 2 C)		[43]

**Table 2:** Characteristic, function, and network structure of multi-dimensional conductive network.

Chemical Formula	Network Structure	Characteristic/ Function	Electrical Conductivity	Discharge Capacity (High Rate)	Ref.
C@LFP/CNTs	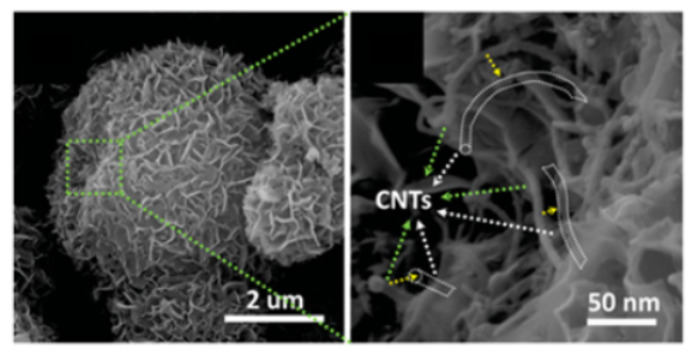	Porous structure/provides favorable kinetics for both electrons and Li^+^	7.71 × 10^−2^ S cm^−1^ (Conductivity of C@LFP/CNTs) 5.91 × 10^−3^ S cm^−1^ (Conductivity of C@LFP)	102 mAh g^−1^ (C@LFP/CNTs at 20 C) 50 mAh g^−1^ (C@LFP at 20 C)	[54]
LFP@C/G	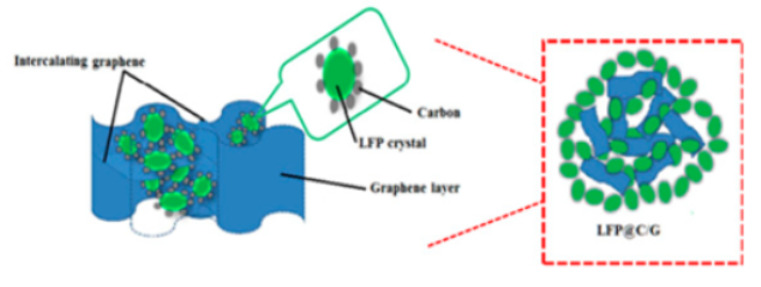	3D “sheets-in-pellets” and “pellets-on-sheets” conducting network structure/highly conductive and plentiful mesopores promote electronic and ionic transport	28.4 Ω (R_ct_ values for LFP@C/G) 75.5 Ω (R_ct_ values for LFP@C)	81.2 mAh g^−1^ (LFP@C/G at 20 C)	[56]
CNT/LFP	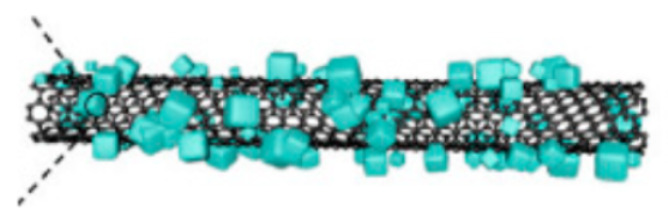	Distinctive loose and scaffolded composite structure/enhances the overall conductivity of the composite	32.47 Ω (R_ct_ values for LFP-CNT-G) 46.23 Ω (R_ct_ values for LFP)	143 mAh g^−1^ (LFP-CNT at 20 C)	[27]
LFP-CNT-G	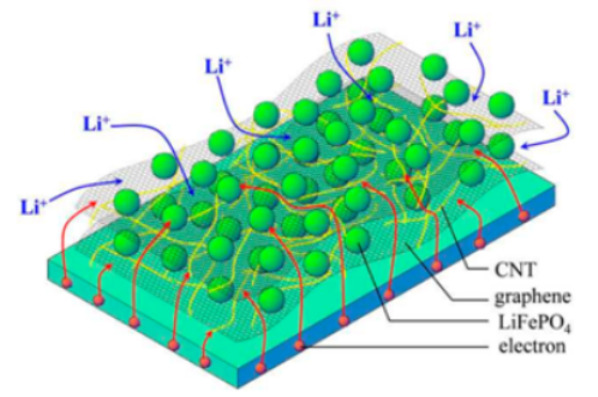	3D conducting networks/faster electron transfer and lower resistance during the Li-ions’ reversible reaction	50.17 Ω (R_ct_ values for LFP-CNT-G) 103.93 Ω (R_ct_ values for LFP-CNT)	115.8 mAh g^−1^ (LFP-CNT-G at 20 C) 99.4 mAh g^−1^ (LFP-CNT at 20 C)	[58]
LFP@C/MXene	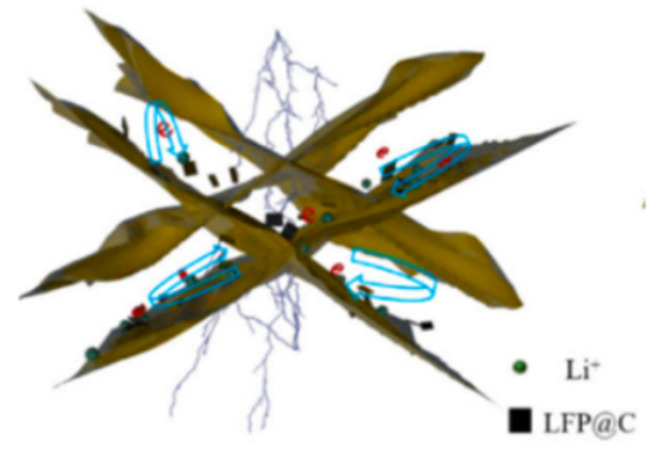	Hierarchically porous structure and “dot-to-surface” conductive network/fast ion and electron transfer for redox reactions	17.26 Ω (R_ct_ values for LFP@C/MX-3.0) 93.32 Ω (R_ct_ values for LFP@C)	140.3 mA h·g^−1^ (LFP@C/MX-3.0 at 20 C) 86.6 mA h·g^−1^ (LFP@C at 20 C)	[59]
LFP/R-GO	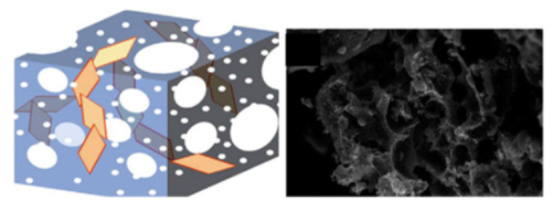	Highly meso-porous structure/good electronic conductivity and high electrolyte permeability	25 Ω (R_ct_ values for LiFePO_4_/R-GO) 50 Ω (R_ct_ values for LiFePO_4_)	135 mAh g^−1^ (LiFePO_4_/R-GO at 5 C)	[60]
LFP/MXene/CNT/Cellulose	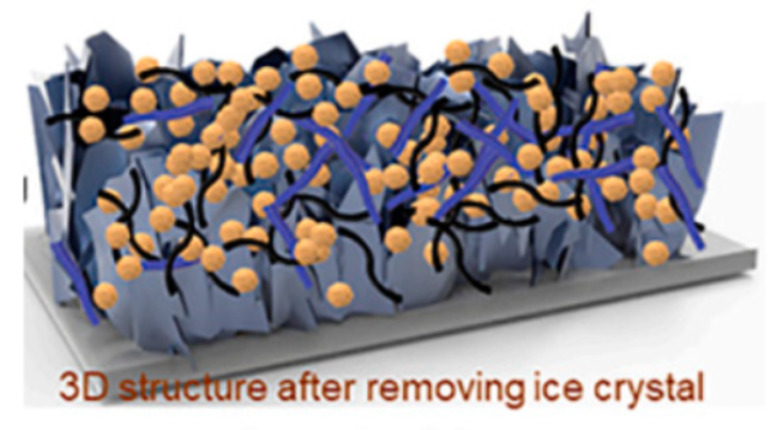	3-dimensional MXene-Carbon nanotubes-Cellulose-LiFePO_4_ (3D-MCC-LFP)/faster electronic/ionic transport	26.6 Ω (R_ct_ values for 3D-MCC-LFP10) 32.2 Ω (R_ct_ values for Con-LFP10)	159.5 mAh g^−1^ (3D-MCC-LFP_120_ at 1 mA cm^−2^)	[61]
LFP/Ti_3_C_2_Tx/CNTs	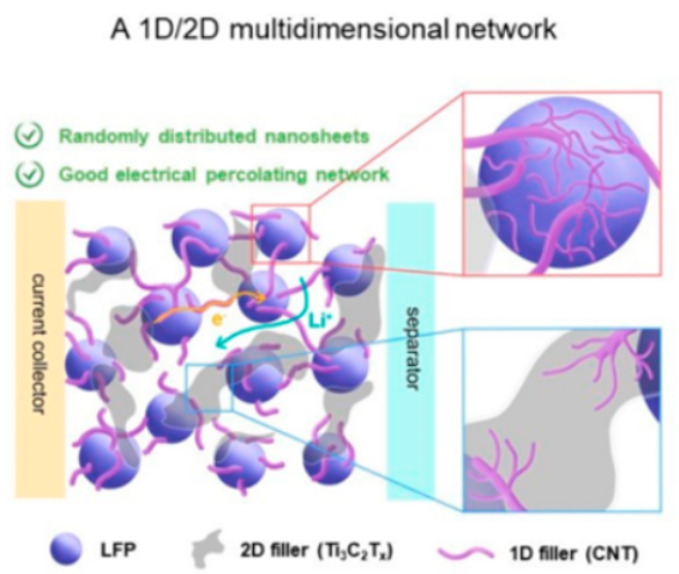	1D single-walled carbon nanotubes (CNTs)/bound together using 2D MXene (Ti_3_C_2_Tx) nanosheets/highlights the ability of multi-dimensional conductive fillers to realize simultaneously superior electrochemical and mechanical properties	27.7 Ω (R_ct_ values for LFP/CNT/Ti_3_C_2_Tx) 32.2 Ω (R_ct_ values for LFP/Ti_3_C_2_Tx)		[62]

## Data Availability

Not applicable.
